# Enzymatic tissue processing after testicular biopsy in non-obstructive azoospermia enhances sperm retrieval

**DOI:** 10.1093/hropen/hoad039

**Published:** 2023-10-18

**Authors:** V Vloeberghs, N De Munck, A Racca, I Mateizel, K Wouters, H Tournaye

**Affiliations:** Centre for Reproductive Medicine, Universitair Ziekenhuis Brussel, Vrije Universiteit Brussel, Brussels, Belgium; Centre for Reproductive Medicine, Universitair Ziekenhuis Brussel, Vrije Universiteit Brussel, Brussels, Belgium; Department of Obstetrics, Gynaecology and Reproductive Medicine, Dexeus University Hospital, Barcelona, Spain; Centre for Reproductive Medicine, Universitair Ziekenhuis Brussel, Vrije Universiteit Brussel, Brussels, Belgium; Centre for Reproductive Medicine, Universitair Ziekenhuis Brussel, Vrije Universiteit Brussel, Brussels, Belgium; Centre for Reproductive Medicine, Universitair Ziekenhuis Brussel, Vrije Universiteit Brussel, Brussels, Belgium

**Keywords:** enzymatic digestion, TESE, non-obstructive azoospermia, sperm retrieval, cumulative live birth delivery

## Abstract

**STUDY QUESTION:**

What is the added value of enzymatic processing of testicular biopsies on testicular sperm retrieval (SR) rates for patients with non-obstructive azoospermia (NOA)?

**SUMMARY ANSWER:**

In addition to mechanical mincing, enzymatic digestion increased SR rates in testicular biopsies of NOA patients.

**WHAT IS KNOWN ALREADY:**

Many studies focus on the surgical approach to optimize recovery of testicular sperm in NOA, and in spite of that, controversy still exists about whether the type of surgery makes any difference as long as multiple biopsies are taken. Few studies, however, focus on the role of the IVF laboratory and the benefit of additional lab procedures, e.g. enzymatic digestion, in order to optimize SR rates.

**STUDY DESIGN, SIZE, DURATION:**

This retrospective single-center cohort study included all patients who underwent their first testicular sperm extraction (TESE) by open multiple-biopsy method between January 2004 and July 2022. Only patients with a normal karyotype, absence of Y-q deletions and a diagnosis of NOA based on histology were included. The primary outcome was SR rate after mincing and/or enzymes. The secondary outcome was cumulative live birth (CLB) after ICSI with fresh TESE and subsequent ICSI cycles with frozen TESE.

**PARTICIPANTS/MATERIALS, SETTING, METHODS:**

Multiple biopsies were obtained from the testis, unilaterally or bilaterally, on the day of oocyte retrieval. Upon mechanical mincing, biopsies were investigated for 30 min; when no or insufficient numbers of spermatozoa were observed, enzymatic treatment was performed using Collagenase type IV. Multivariable regression analysis was performed to predict CLB per TESE by adjusting for the following confounding factors: male FSH level, female age, and requirement of enzymatic digestion to find sperm.

**MAIN RESULTS AND THE ROLE OF CHANCE:**

We included 118 patients, of whom 72 (61.0%) had successful SR eventually. Spermatozoa were retrieved after mechanical mincing for 28 patients (23.7%; 28/118) or after additional enzymatic digestion for another 44 patients (37.2%; 44/118). Thus, of the 90 patients requiring enzymatic digestion, sperm were retrieved for 44 (48.9%). Male characteristics were not different between patients with SR after mincing or enzymatic digestion, in regard to mean age (34.5 vs 34.5 years), testis volume (10.2 vs 10.6 ml), FSH (17.8 vs 16.9 IU/l), cryptorchidism (21.4 vs 34.1%), varicocele (3.6 vs 4.6%), or histological diagnosis (Sertoli-cell only 53.6 vs 47.7%, maturation arrest 21.4 vs 38.6%, sclerosis/atrophy 25.0 vs 13.6%).

Of the 72 patients with sperm available for ICSI, 23/72 (31.9%) achieved a live birth (LB) after the injection with fresh testicular sperm (and fresh or frozen embryo transfers). Of the remaining 49 patients without LB, 34 (69.4%) had supernumerary testicular sperm frozen. Of these 34 patients, 19 (55.9%) continued ICSI with frozen testicular sperm, and 9/19 (47.4%) had achieved an LB after ICSI with frozen testicular sperm. Thus, the total CLB was 32/118 (27.1%) per TESE or 32/72 (44.4%) per TESE with sperm retrieved.

Of the female characteristics (couples with sperm available), only female age (30.3 vs 32.7 years; *P* = 0.042) was significantly lower in the group with a LB, compared to those without.

The CLB with testicular sperm obtained after enzymatic digestion was 31.8% (14/44), while the CLB with sperm obtained after mincing alone was 64.3% (18/28). Multivariable logistic regression analysis showed that when enzymatic digestion was required, it was associated with a significant decrease in CLB per TESE (OR: 0.23 (0.08–0.7); *P* = 0.01).

**LIMITATIONS, REASONS FOR CAUTION:**

Limitations of the study are related to the retrospective design. However, the selection of only patients with NOA, and specific characteristics (normal karyotype and absence Y-q deletion) and having their first TESE, strengthens our findings.

**WIDER IMPLICATIONS OF THE FINDINGS:**

Enzymatic processing increases the SR rate from testicular biopsies of NOA patients compared to mechanical mincing only, demonstrating the importance of an appropriate laboratory protocol. However, NOA patients should be counseled that when sperm have been found after enzymatic digestion, their chances to father a genetically own child may be lower compared to those not requiring enzymatic digestion.

**STUDY FUNDING/COMPETING INTEREST(S):**

None reported.

**TRIAL REGISTRATION NUMBER:**

N/A.

WHAT DOES THIS MEAN FOR PATIENTS? Men with no sperm cells (spermatozoa) in their ejaculate (a condition called ‘azoospermia’) can father children by having a testicular biopsy performed (in which a small piece of testicular tissue is surgically removed) to retrieve spermatozoa from the testis, before IVF. If the azoospermia results from an obstruction of the sperm ducts, i.e. in the epididymis or vas deferens, spermatozoa will be retrieved from the testis in all men. However, in men where the production of spermatozoa is impaired, i.e. in a condition known as ‘non-obstructive azoospermia’, sperm will only be found in around half of them.

In order to retrieve spermatozoa, after performing a testicular biopsy, the procured tissue is handled in the laboratory. The tissue is microscopically examined after mincing the material into smaller pieces to facilitate the retrieval of spermatozoa. Eventually a second step, involving the use of enzymatic digestion to digest the surrounding tissue of the biopsy, can improve both the visualization and eventually retrieval of spermatozoa. However, not all laboratories use this extra step, and therefore, this study investigates whether this ‘enzymatic digestion step’ can optimize the reproductive outcome in patients with non-obstructive azoospermia.

We conclude that the use of enzymatic digestion facilitates the retrieval of spermatozoa and can thus be useful to help men with non-obstructive azoospermia to have their genetically own child.

## Introduction

Testicular sperm extraction (TESE) in combination with ICSI has been a major breakthrough in the management of men with non-obstructive azoospermia (NOA), with the first children born following this procedure in 1995 (Devroey *et al.*, 1995; Tournaye *et al.*, 1995). However, to date, only a proportion of men with NOA can have their genetically own child because of limitations in both sperm retrieval (SR) rates and outcomes after ICSI when using their testicular sperm (Vloeberghs *et al.*, 2015; Corona *et al.*, 2019).

Sperm can be retrieved in virtually all men with obstructive azoospermia (OA), whereas without preliminary patient selection, this will only be the case in about half of men with NOA, with the SR rate varying from 17% to 80% depending on patient selection (Donoso *et al.*, 2007; Corona *et al.*, 2019). Accurate diagnosis of NOA is based on histopathology. Inclusion of patients with only a clinical diagnosis of azoospermia (without testicular histology) or patients with hypospermatogenesis may result in overestimation of SR rates due to inclusion of misdiagnosed OA (Matsumiya *et al.*, 1994; Tournaye, 2001).

Several factors (testicular volume, FSH level, and inhibin B) have been investigated as predictors of SR in men with NOA (Tournaye *et al.*, 1997; Vernaeve *et al.*, 2004; Adamopoulos and Koukkou, 2010; Chen *et al.*, 2010; Toulis *et al.*, 2010; Bryson *et al.*, 2014; Yildirim *et al.*, 2014; Yang *et al.*, 2015), and have been introduced in different prediction models (Bernie *et al.*, 2013; Boitrelle *et al.*, 2011; Cissen *et al.*, 2016; Tournaye, 2011), yet all with disappointing results. Only preliminary testicular histology has some value in predicting sperm recovery (Boitrelle *et al.*, 2011; Tournaye, 2011; Abdel Raheem *et al.*, 2013). The routine genetic work-up with screening for the presence of Yq microdeletions may also guide treatment, as patients with a deletion of the AZFa or AZFb region will have no sperm production (Stouffs *et al.*, 2017; Minhas *et al.*, 2021).

Two surgical approaches are described for SR in NOA: multiple-biopsy conventional TESE (c-TESE) or microsurgical TESE (m-TESE). Although in general it is assumed that the latter approach may increase the SR (Bernie *et al.*, 2015; Minhas *et al.*, 2021), notwithstanding the lack of well-designed controlled trials, correct interpretation of the existing evidence does not show superiority for either approach over conventional TESE (Van Peperstraten *et al.*, 2008; Deruyver *et al.*, 2014; Corona *et al.*, 2019, 2020).

Surprisingly, the additional role of the ART laboratory procedures after surgical SR for NOA has been rather underdiscussed. Since testicular samples contain a large number of different cell types, especially red blood cells, and given that the elongated spermatids attached within the seminiferous tubules have to be released from these tubules, a number of laboratory methods can be applied to improve the observation of spermatozoa and, eventually, sperm recovery.

The methods described in the literature include mechanical mincing and shredding of the testicular tissue with fine needles (Verheyen *et al.*, 1995), application of erythrocyte-lysing buffer (ELB) (Nagy *et al.*, 1997), and the enzymatic digestion method (Crabbé *et al.*, 1997). Shredding and mincing is the most widely used first step, and often the sole step, for SR in the laboratory as it aims to rupture the testicular tissue and spread the seminiferous tubules (Verheyen *et al.*, 2017). Red blood cells present in the testicular tissue may interfere with proper visualization of spermatozoa in the suspension. ELB lyses the red blood cells in the suspension and improves visualization, especially when spermatozoa are immotile (Nagy *et al.*, 1997), although this is not without risk (Yazdinejad *et al.*, 2020). In NOA, sperm production is limited by very low numbers of spermatozoa and only scarce testicular tissue can be removed. This is complicating the retrieval of spermatozoa after mechanical searches and it is time consuming for the lab, when compared to samples taken from patients with OA. Enzymatic digestion of the cellular matrix may improve visualization of sperm hidden between other cells, facilitating sperm recovery (Verheyen *et al.*, 2017). Both Collagenase type IA and IV have been used to digest collagen of the basal membrane and extracellular matrix (Salzbrunn *et al.*, 1996; Crabbé *et al.*, 1997). Crabbé *et al.* (1997) have studied four different types of collagenases and found type IV to be the most efficient in terms of sperm recovery. Only a few studies focus on the benefit of enzymatic tissue digestion to improve SR when no spermatozoa have been found after mechanical mincing. These studies report SR rates after supplementary enzymatic digestion of 26.9% (7/27); 33.0% (37/112) and 7.0% (35/501), respectively (Crabbé *et al.*, 1998; Aydos et al. 2005; Ramasamy *et al.*, 2011). Although these studies illustrate a rationale to adopt enzymatic treatment, most ART clinics still use the mechanical method alone or do not mention any additional processing in the lab (Corona *et al.*, 2019).

Therefore, the aim of the present retrospective study is to assess the added value of enzymatic digestion to mechanical mincing on SR after TESE in a cohort of strictly defined NOA cases based on histological diagnosis of a random biopsy taken during the TESE procedure itself.

Spermatozoa found after additional enzymatic digestion are more difficult to recover and possibly this may affect the outcome of ICSI. Therefore, we also compared the results of ICSI with testicular sperm obtained after mincing and enzymatic digestion to those obtained after mincing alone, by calculating the cumulative live birth (CLB) delivery rates after ICSI cycles with fresh TESE and subsequent ICSI cycles with frozen TESE.

## Materials and methods

### Study design and patients

In this retrospective single-center cohort study, we included all azoospermic patients who had their very first TESE by open biopsy between January 2004 and July 2022. Patients who had TESE previously, in our hospital or elsewhere, were excluded. All patients had a work-up with standard clinical examination, endocrine profile (FSH, luteinizing hormone (LH) and testosterone) and genetic analysis (karyotype and Yq deletion screening). Over the whole study period, a scrotal ultrasound was performed in patients with a history of cryptorchidism. No major abnormal ultrasound findings that could interfere with our results were detected. Across the study period, testicular volumes were consistently measured by a Prader orchidometer.

Only patients with a normal karyotype (46, XY) and absence of Yq deletions were included. As the World Health Organization advises that the diagnosis of NOA should be based on the histopathological findings (Tournaye, 2001), only NOA patients with severely impaired or incomplete spermatogenesis as shown by histology were included. The classification as proposed by Levin (1979) was used, where normal spermatogenesis refers to spermatogenic activity of the normal postpubertal male. Hypospermatogenesis refers to a generalized reduction in the number of spermatogenic cells within the seminiferous tubules. Maturation arrest refers to the condition in which spermatogenesis is mainly arrested at the primary spermatocyte stage. Incomplete maturation arrest is characterized by the presence of focal seminiferous tubules containing elongated spermatids or spermatozoa. Germ-cell aplasia refers to seminiferous tubules which are completely devoid of germinal cells and lined exclusively by Sertoli cells, i.e. Sertoli-cell only (SCO) syndrome. Incomplete germ-cell aplasia refers to the presence of rare foci of complete spermatogenesis in combination with Sertoli-cell only tubules. Tubular sclerosis refers to seminiferous tubules showing reduced to absent spermatogenesis with thickening of the tunica propria progressing to sclerosis. The testicular histology of the included patients showed either maturation arrest with or without focal spermatogenesis, germ-cell aplasia (SCO) with or without focal spermatogenesis, or tubular sclerosis and atrophy with or without focal spermatogenesis. While there was no histological preselection of ‘good prognosis’ patients in our analysis, histopathology obtained after SR was used in retrospect to overcome potential selection bias associated with the known errors after pure clinical diagnosis of NOA (Matsumiya *et al.*, 1994).

Men with a histological diagnosis of hypospermatogenesis were excluded from the analysis as they have an SR rate similar to patients with normal spermatogenesis (Matsumiya *et al.*, 1994), therefore making redundant any add-on procedure aiming to improve SR rates.

### Testicular biopsy

Patients had an open testicular biopsy under general anesthesia or occasionally under local anesthesia. The TESE was performed as a therapeutic procedure and planned on the day of oocyte retrieval for ICSI. In our setting, the operating theater is located next to the IVF laboratory and direct communication with the laboratory staff is possible during the procedure.

Multiple testicular biopsies were taken from the testis unilaterally or bilaterally. Bilateral sampling was performed when sperm recovery after wet preparation (mechanical dispersion) of the first testis in the laboratory was communicated as negative. Up to six biopsies were obtained, depending on the volume of the testes. To date, there is ample evidence that the surgical recovery technique does not interfere with recovery rates as long as multiple biopsies are taken (Corona *et al.*, 2019, 2020). Therefore, the m-TESE procedure was performed according to Schlegel on patient request, with testicular biopsies taken under magnification 15×–20× targeting the spots where tubules were more dilated (Schlegel, 1999). A randomly taken biopsy of each testis was sent for histological diagnosis. The remaining testicular biopsies were collected and placed in a Petri dish with HEPES-buffered medium and handed over to the adjacent IVF laboratory.

### Laboratory procedures

The first laboratory step was the mechanical method or mincing. Tissue was finely minced, and tubules were teased apart with sterile 18 G needles in order to facilitate the release of sperm. The cell suspension was microscopically evaluated (200× or 400×) and feedback was given to the operation room regarding the presence or absence of sperm. If spermatozoa were observed, the minced tissue was centrifuged at 800*g* for 5 min and the pellet was searched for the presence of mature-looking sperm or elongated spermatids under 400× magnification (Verheyen *et al.*, 1995).

In case of high concentrations of erythrocytes, ELB was used to enhance visualization of spermatozoa (Nagy *et al.*, 1997). Testicular sperm pellets were resuspended in 2–4 ml of ELB (155 mM NH_4_Cl, 10 mM KHCO_3_, and 2 mM ethylenediaminetetraacetic acid) for 10 min at room temperature. The reaction was stopped by adding 10 ml of HEPES-buffered medium. After centrifugation, 10 µl droplets of the pellet were placed in a Petri dish overlayered with oil and searched for spermatozoa.

In any cases with no or insufficient number and/or quality of spermatozoa observed after a standardized protocol of 30 min search following mechanical mincing, enzymatic digestion was performed using Collagenase type IV. Residual tissue pieces were centrifuged, and the pellet was resuspended in 1 ml of the Collagenase IV solution. Initially, the solution was in-house prepared: HEPES-buffered medium supplemented with 5% HSA, 1.6 mM CaCl_2_ (Merck, Darmstadt, Germany), 25 µl/ml DNAse (Sigma Chemical Co., St Louis, MO, USA, Crude DN25), and 1000 IU/ml Collagenase type IV (Sigma C5138), pH 7.2. But since 2013, a switch was made to the commercially available, ready-to-use Collagenase solution (GM501 Collagenase^®^, Gynemed, Germany). The suspension was incubated for 1 h at 37°C to allow digestion (Crabbé *et al.*, 1997; Verheyen *et al.*, 2017). The samples were shaken every 5–10 min during incubation. After 1 h, the suspension was diluted with 10 ml HEPES-buffered medium and centrifuged at 800*g* for 5 min. The pellet was resuspended, and 10 µl droplets of the pellet were placed in a Petri dish, overlayered with oil, and microscopically examined by the embryologist at 200× to 400× magnification.

Spermatozoa were used for injection of mature (MII) oocytes on the day of the oocyte retrieval procedure. The testicular cell suspension was frozen for later use if supernumerary spermatozoa were still available after injection of all MII oocytes (Verheyen *et al.*, 2004).

### Outcome measures

The primary outcome was the SR rate to perform ICSI. The testicular cell suspension was considered positive, if at least one, preferably motile, spermatozoon was observed for ICSI. Fully elongated spermatids and immature-looking spermatozoa were both categorized as ‘spermatozoa’ because it is impossible to make a distinction between both types after preparation. The testicular suspension was considered negative if no sperm or only immature germ cells were observed.

Female partners had ovarian stimulation with recombinant or urinary FSH in combination with gonadotrophin releasing hormone (GnRH) agonist or antagonist. Oocyte retrieval was scheduled on the same day as the TESE. Motility and morphology of the sperm used for ICSI was reported. Fertilization was assessed 16–18 h after injection by the presence of two pronuclei (2PN). Embryos were scored according to their morphological appearance (De Munck *et al.*, 2015). Up to three (exceptionally four) embryos were transferred into the uterine cavity on Day 3 or Day 5 after injection. Since July 2003, the number of embryos for transfer was restricted by the Belgian law (Van Landuyt *et al.*, 2006). Supernumerary embryos of sufficient quality were cryopreserved for later use. The utilization rate was calculated based on all embryos that were transferred or cryopreserved on Day 3 or Day 5/6. A clinical pregnancy was defined by the presence of a gestational sac at transvaginal ultrasound 5 weeks after embryo transfer. Patients with unknown outcome were considered to be not pregnant. A live birth (LB) was defined as live infant of at least 20 weeks of gestational age or with a birthweight of >500 g (Bonduelle *et al.*, 2002).

The secondary outcome was CLB after ICSI with fresh TESE and subsequent ICSI cycles with frozen TESE. When patients delivered following transfer of frozen embryos, this LB was added to the result of the (unsuccessful) fresh ICSI cycle. When patients still had frozen testicular sperm after a first (unsuccessful) ICSI cycle with fresh testicular sperm, the LBs from subsequent ICSI cycles with frozen testicular sperm were added to give the total CLB after fresh and frozen TESE.

### Statistical analysis

Patients were categorized into two groups according to finding (SR positive) or not finding (SR negative) spermatozoa during the therapeutic TESE. Comparisons between groups were performed by mean of Pearson’s chi-squared test, Fisher’s exact test and Mann Whitney *U*-test, respectively, for categorical and continuous variables. Furthermore, the CLB was calculated per TESE procedure whenever spermatozoa were found. Finally, a multivariable logistic regression was performed where the dependent variable was CLB and the independent variables were enzymatic digestion, male FSH, and female age. All variables were simultaneously entered in the logistic regression model. All statistical tests used a two-tailed *α* 0.05. Analyses were performed using STATA^®^ 15.1 (Statistical Software: Release 15, College Station, TX, USA: StataCorp LL).

### Institutional review board approval

The study was approved by the local institute’s Ethics Committee (number 2020-186). According to Belgian legislation, all data of patients were fully anonymized before analysis.

## Results

**Figure 1.**
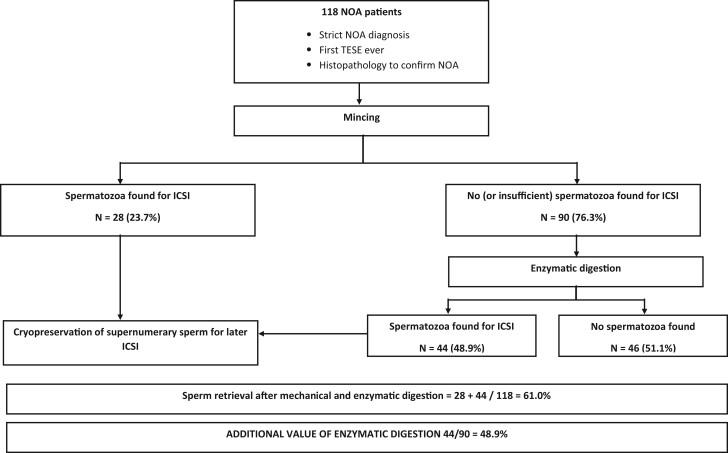
**Schematic overview of patients included in the study.** NOA, non-obstructive azoospermia; TESE, testicular sperm extraction; N, number of patients; ICSI, intracytoplasmic sperm injection.

In total, 118 NOA patients were included, with an overall SR of 61.0%. For 28 men (23.7%), spermatozoa were found after simple mechanical mincing and a standardized 30-min observation. For another 44 patients (37.2%; 44/118), spermatozoa were retrieved after additional enzymatic digestion of the biopsy. Therefore, of the 90 patients who had no sperm following mechanical mincing, 48.9% (44/90) had sperm retrieved after enzymatic digestion (Fig. 1).

No difference in mean FSH, mean age, mean testicular volume, histological diagnosis of SCO or sclerosis and/or atrophy or maturation arrest, history of cryptorchidism or presence of varicocele was observed between patients with sperm retrieved or not (Table 1). Similarly, no difference in the above parameters was found between patients for whom sperm was found after mincing or after enzymatic digestion (Table 2).

**Table 1.** Patient characteristics according to outcome of sperm retrieval after TESE.

	Positive SR (N = 72)	Negative SR (N = 46)	*P*-value
FSH (mean ± SD) IU/l	17.3 ± 10.1	20.1 ± 11.7	0.193
Male Age (mean ± SD) y	35.1 ± 6.5	36.6 ± 6.5	0.204
Volume testis (mean ± SD) ml	10.5 ± 4.1	9.9 ± 3.8	0.468
Histopathology			
SCO (%)	36 (50.0)	32 (69.6)	0.054**
MA (%)	23 (31.9)	6 (13.0)	
Sclerose and/or atrophy (%)	13 (18.1)	8 (17.4)	
Cryptorchidism (%)	21 (29.2)	10 (21.7)	0.371*
Varicocele (%)	3 (4.2)	1 (2.1)	0.492**

TESE, testicular sperm extraction; SR, sperm retrieval; FSH, follicle-stimulating hormone; y, years; ml, milliliter; SCO, Sertoli-cell only; MA, maturation arrest.

* Pearson’s chi squared test.

** Fisher’s exact test.

**Table 2.** Patient characteristics according to testicular sperm retrieved after mincing only or after enzymatic digestion.

	Sperm found after mincing (N = 28)	Sperm found after enzymatic digestion (N = 44)	*P*-value
FSH (mean ± SD) IU/l	17.8 ± 7.9	16.9 ± 11.4	0.701
Male Age (mean ± SD) y	34.5 ± 5.9	35.4 ± 6.9	0.589
Volume testis (mean ± SD) ml	10.2 ± 3.8	10.6 ± 4.3	0.639
Histopathology			
SCO (%)	15 (53.6)	21 (47.7)	0.231**
MA (%)	6 (21.4)	17 (38.6)	
Sclerose and/or atrophy (%)	7 (25)	6 (13.6)	
Cryptorchidism (%)	6 (21.4)	15 (34.1)	0.188**
Varicocele (%)	1 (3.6)	2 (4.6)	0.999**

FSH, follicle-stimulating hormone; y, years; ml, milliliter; SCO, Sertoli-cell only; MA, maturation arrest.

** Fisher’s exact test.

Table 3 illustrates embryology and LB data of 72 ICSI cycles with fresh testicular sperm retrieved after mincing (n = 28) or after enzymatic digestion (n = 44). The fertilization rate was significantly lower in the group where sperm had been found after enzymatic digestion (63.7% vs 41.4%; *P* = 0.002). The number of cycles with total fertilization failure was lower, but not significantly different, when sperm had been found following enzymatic digestion rather than mincing (3.6% vs 15.9%; *P* = 0.087). On Day 3, embryo quality per 2PN oocyte was comparable between the cycles with sperm retrieved after mincing or after enzymatic digestion for all three categories: excellent, good, and moderate (*P* = 0.068, *P* = 0.589, and *P* = 0.106, respectively). The utilization rates per MII oocyte and per 2PN oocyte were not significantly different between the two groups (*P* = 0.216 and *P* = 0.410, respectively). The number of cycles with insufficient embryo quality for embryo transfer or for cryopreservation was comparable between the cycles with sperm retrieved after mincing or after enzymatic treatment. We observed a higher prevalence of injection with immotile and morphologically abnormal sperm if spermatozoa had been retrieved after enzymatic digestion (data not shown).

**Table 3.** Embryology and live birth data between patients with testicular sperm retrieved after mincing only or after enzymatic digestion.

	Sperm found after mincing (N = 28)	Sperm found after enzymatic digestion (N = 44)	*P*
Female age (mean ± SD) y	31.4 ± 5.0	31.8 ± 5.0	0.732
Number of COCs (mean ± SD)	11.8 ± 7.2	13.1 ± 8.5	0.508
Number of MII oocytes (mean ± SD)	9.3 ± 5.2	10.5 ± 6.9	0.418
Injected MII oocytes (mean ± SD)*	9.1 ± 5.2	8.8 ± 6.2	0.786
Fertilization			
2PN oocytes (mean ± SD)	5.8 ± 4.4	3.8 ± 3.7	**0.05**
Fertilization rate per injected MII oocyte (%)	63.7	41.4	**0.002**
Cycles without fertilization (%)	1/28 (3.6)	7/44 (15.9)	0.087^°^
Day 3 embryo quality			
Excellent (mean ± SD)	2.5 ± 1.8	3.2 ± 1.7	0.068^#^
Good (mean ± SD)	2.4 ± 1.9	1.9 ± 1.0	0.589^#^
Moderate (mean ± SD)	2.3 ± 1.7	1.8 ± 1.3	0.406^#^
Cycles with insufficient EQ (%)	1/28 (3.6)	8/44 (18.2)	0.069^#^
Utilization rate/MII oocyte (mean ± SD)	37.8 ± 22.7	30.5 ± 22.4	0.216
Utilization rate/2PN oocyte (mean ± SD)	61.1 ± 31.7	67.9 ± 34.6	0.410
Live birth rate after fresh TESE (%)	10/28 (35.7)	13/44 (29.6)	0.595
Total CLB rate after fresh + frozen TESE (%)	18/28 (64.3)	14/44 (31.8)	**0.007**

y, years; COC, cumulus oocyte complex; MII oocyte, metaphase II (mature) oocytes; EQ, embryo quality; ET, embryotransfer; CLB, cumulative life birth; TESE, testicular sperm extraction; 2PN, two-pronuclear (fertilized) oocytes. Bold values indicate statistical significance at the p < 0.05 level.

* Only oocytes injected with testicular sperm are presented.

° Fisher’s exact test.

# Mann Whitney test.

**Figure 2.**
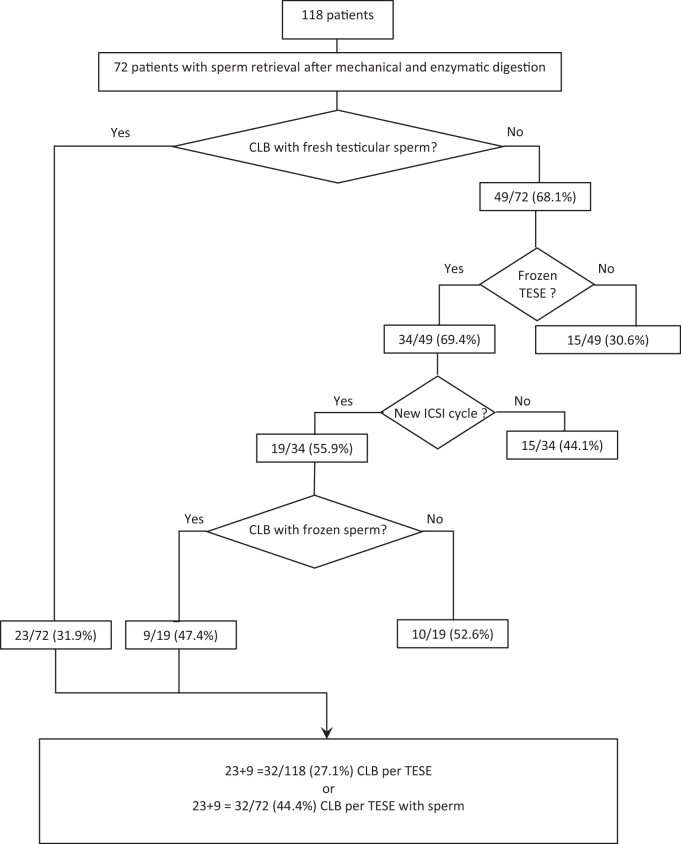
**Schematic overview of pathways leading to live birth births after ICSI with fresh and/or frozen testicular sperm.** CLB, cumulative live birth; ICSI, intracytoplasmic sperm injection; TESE, testicular sperm extraction.

Of the 72 patients with sperm available for ICSI, 23/72 (31.9%) achieved an LB after the injection with fresh testicular sperm and embryo transfer (of fresh and frozen embryos), while 9/19 (47.4%) achieved an LB after the use of frozen testicular semen (Fig. 2). The CLB with fresh testicular sperm appeared to be lower when enzymatic digestion was required to find sperm (35.7% vs 29.6%; *P* = 0.595) (Table 3). There were 49 patients remaining without LB, of whom 34 had supernumerary testicular sperm frozen. The other 15 patients did not have supernumerary sperm to freeze. Of the 34 patients with frozen testicular sperm, 19 had one or more ICSI cycles with frozen testicular sperm. Eleven of these 19 patients had frozen testicular sperm found after mincing, and so 25 ICSI cycles and 22 embryo transfers (of fresh or frozen embryos) were performed. Eight of the 19 patients had frozen testicular sperm found after enzymatic digestion, allowing 10 ICSI cycles and 17 embryo transfers (of fresh and frozen embryos). Of the 19 patients performing ICSI with frozen testicular sperm, 9/19 (47.4%) had an LB after ICSI with frozen testicular sperm, giving rise to a total CLB of 32/118 (27.1%) per TESE or 32/72 (44.4%) per TESE with sperm retrieved (Fig. 2). The total CLB of ICSI cycles with fresh and frozen testicular sperm was significantly lower when enzymatic digestion was required to find sperm (31.8% vs 64.3%; *P* = 0.007) (Table 3).

Looking to the female and male characteristics (of couples with sperm available) and technique used in the laboratory (mincing alone vs enzymatic digestion), only female age (30.3 vs 32.7 years—*P* = 0.042) and the proportion of patients using enzymatic digestion to find sperm (43.6% vs 75.0%—*P* = 0.008) were significantly lower in the group with an LB after TESE (Table 4).

**Table 4.** Comparison of the female, male characteristics (of couples with sperm available), and the technique used is the laboratory to find sperm of patients having a live birth (CLB per TESE positive) or having no live birth (CLB per TESE negative) after the use of fresh and frozen testicular sperm.

	Live Birth (N = 32)	No Live Birth (N = 40)	*P*
**Female characteristics**			
Age (mean ± SD) y	30.3 ± 4.4	32.7 ± 5.2	**0.042** ^°^
BMI (mean ± SD)	24.6 ± 4.3	24.8 ± 5	0.856
Number of COC (mean ± SD)	12.7 ± 7.3	12.5 ± 8.6	0.555^°^
Total units of FSH (mean ± SD)	1730.9 ± 673.4	2042.3 ± 898.3	0.099^°^
Female factor of infertility	2/28 (7.1)	7/39 (17.9)	0.285
**Male characteristics**			
Age (mean ± SD) y	33.8 ± 5.3	36.1 ± 7.3	0.124
FSH (mean ± SD) IU/l	16.9 ± 8.1	17.5 ± 11.7	0.930
Volume testis (mean ± SD) ml	10 ± 3.1	10.8 ± 4.8	0.644
Histopathology			
SCO (%)	17/36 (47.2)	19/39 (52.8)	0.822
MA (%)	9/23 (39.1)	14/23 (60.9)	
Sclerose and/or atrophy (%)	6/13 (46.2)	7/13 (53.9)	
**Laboratory technique**			
Enzymatic digestion (%)	14/32 (43.6)	30/40 (75.0)	**0.008**

FSH, follicle-stimulating hormone; y, years; ml, milliliter; SCO, Sertoli-cell only; MA, maturation arrest; COC, cumulus oocyte complex; CLB, cumulative live birth rate; TESE, testicular sperm extraction; BMI, body mass index. Bold values indicate statistical significance at the p < 0.05 level.

° Non-parametric Ranksum.

Multivariable logistic regression analysis showed that enzymatic digestion was associated with a significant reduction in the CLB per TESE, while male FSH and female age were not (Table 5).

**Table 5.** Multivariable logistic regression was performed to predict total CLB rate after the use of fresh and frozen testicular sperm for the confounding factors listed in the table.

	Odds ratio	*P*-value	95% CI	
Male FSH	0.99	0.962	0.94	1.06
Enzymatic digestion required to find sperm	0.23	**0.01**	0.08	0.7
Female AGE	0.92	0.167	0.8	1.03

CLB, cumulative live birth rate; FSH, follicle-stimulating hormone. Bold values indicate statistical significance at the p < 0.05 level.

## Discussion

In this large single-center retrospective study in a well-defined NOA population without proper preselection of good prognosis patients by previous testicular biopsy or histopathology, the overall SR rate was 61.0% which is in accordance with SR rates reported in a recent meta-analysis (Corona *et al.*, 2019). In contrast to studies focusing on the surgical technique for facilitating SR, only a few studies have investigated the added value of laboratory procedures, specifically enzymatic digestion, to facilitate SR. Unfortunately, as indicated by the authors of the above-mentioned meta-analysis, insufficient data are available in the literature to evaluate the added value of enzymatic digestion for SR.

Here, we present a large study addressing this issue and we demonstrate the added value of enzymatic digestion to simple mechanical mincing for improving SR in our setting. We found a higher added value of enzymatic digestion compared to previous studies where sperm was retrieved after enzymatic digestion in, respectively, 26.9%, 33.0%, and 7.0% of failures after mechanical mincing (Crabbé *et al.*, 1998; Aydos *et al.*, 2005; Ramasamy *et al.*, 2011). These differences may be explained by differences in sample size (Crabbé *et al.*, 1998), longer searching time after mincing (Crabbé *et al.*, 1998), or patient selection, e.g. inclusion of patients with hypospermatogenesis or patients without further histological assessment (Aydos *et al.*, 2005; Ramasamy *et al.*, 2011). In the larger study by Ramasamy *et al.* (2011), the difference was most obvious, with an added value of enzymatic digestion that was limited to only 7.0%. The inconsistency of our study with this latter study may also be explained by the differences in patient selection, e.g. patients were not necessarily having their first testicular biopsy.

In the present study, the results of SR after mincing (23.7%) are in line with previous studies of Aydos *et al.* (2005) and Crabbé *et al.* (1998). In the study of Ramasamy *et al.* (2011), a higher SR rate after mincing (52.5%) was reported. Again, this may be explained by a different patient selection as discussed above. In our study, only ‘first ever TESE’ patients were included, eliminating any bias by preselection of good-prognosis patients based on previous biopsy and only patients with diagnosis of NOA strictly based on histology.

Another important difference with the study of Ramasamy *et al.* (2011) is the timing when the enzymatic digestion is performed. In our study, enzymatic digestion with Collagenase type IV was performed on the same day of the TESE procedure. However, in this study of Ramasamy *et al.*, if no sperm was retrieved after mincing, the tissue was kept overnight, and enzymatic digestion followed only the next day. This longer incubation time is potentially associated with increased extracellular oxidative stress and possible negative effects on the DNA and fertilizing capacity of the retrieved sperm (Cheng *et al.*, 2022).

Better visualization of spermatozoa after enzymatic digestion increases the chance of selecting better-quality sperm in terms of motility and morphology for ICSI. Nevertheless, the ‘quality’ of the sperm is also a potential concern, in terms of functionality of the sperm obtained after enzymatic digestion, as they are lower in number and more difficult to recover (potentially referring to a more severe degree of primary testicular failure in general).

Whether enzymatic digestion may have a negative effect on fertilization and embryo quality was also investigated in this study. We reported a higher incidence of oocytes injected with immotile and abnormal morphologically sperm if enzymatic digestion was required to find sperm. This was translated in lower fertilization rates and a higher incidence of cycles with failed fertilization after enzymatic digestion. However, once fertilized, embryo development was not affected by the SR technique. A retrospective multi-center study in Germany concluded that the availability of motile testicular sperm for injection was more important than the method used in the laboratory (mechanical method or enzymatic digestion) for fertilization and embryo development (Baukloh *et al.*, 2002). Other studies reported no significant difference in fertilization and embryo quality if testicular sperm retrieved after enzymatic digestion was used for injection (Crabbé *et al.*, 1998; Aydos *et al.*, 2005; Ramasamy *et al.*, 2011).

From the patient’s perspective, achieving a pregnancy and especially having their genetically own child is of utmost importance. Therefore, we calculated, in this study, the CLB rate after fresh TESE including the results of frozen embryos (Fig. 2). The LB rate with fresh TESE was not different if enzymatic digestion was required to find sperm (29.6% vs 35.7%; *P* = 0.595) (Table 3). The only study reporting on LB after ICSI with fresh testicular sperm retrieved after enzymatic digestion was also unable to show a difference in LB whether or not spermatozoa were retrieved after mechanical mincing or enzymatic digestion (Ramasamy *et al.*, 2011). Of the 49 remaining patients without an LB after the first cycle, 34 (69.4%) had still frozen testicular sperm (Fig. 2). We therefore also calculated the CLB rate per TESE including all ICSI cycles with frozen testicular sperm. Not surprisingly, we found that the total CLB rate with fresh and frozen testicular sperm was significantly lower following enzymatic digestion (31.8% vs 64.3%; *P* = 0.007) (Table 3). Indeed, the multivariable logistic regression confirmed the use of enzymatic digestion as the only parameter associated with a lower CLB rate per positive TESE (with sperm retrieved) (Table 4). This is probably because fewer patients were able to freeze sperm for subsequent ICSI cycles if enzymatic digestion was required to retrieve sperm. A second reason may be that the quality (total numbers and motility) of the sperm available for freezing after enzymatic digestion was lower and maybe even more prone to DNA fragmentation post-thawing, resulting in reduced post-thaw fertilization and developmental capacity. Patients for whom enzymatic digestion had been required to find sperm should thus be counseled that they have prognostically a lower chance of fathering their genetically own child after a single TESE procedure. Our follow-up study on children (n = 724) born after using non-ejaculated sperm did not show any difference regarding the neonatal outcome between children of fathers with NOA or OA (Belva *et al.*, 2011). However, to the best of our knowledge, no reports on neonatal outcome of children born of fathers with NOA have made a distinction between sperm being recovered after enzymatic digestion or mincing.

The strength of the present study is that it is a large study in an NOA population strictly defined by testicular histology and without any proper preselection of good prognosis patients aiming at SR for ICSI. Although histology (e.g. degree of germ cell aplasia) correlates with surgical retrieval rates (Tournaye *et al.*, 1997), such a correlation was not subject of this study. The sperm recovery procedure in the laboratory remained independent of the histopathology, with the latter only known post-hoc. However, limitations are inherent to the retrospective design of the study. Although we feel that the enzymatic step following the 30-min search of the wet preparation may also save a significant amount of time, the current design did allow us to conclude whether this was indeed the case. Further, enzymatic digestion in the lab could be proposed as a treatment option for men with a previously failed TESE.

Overall, we may conclude that enzymatic digestion, in addition to mechanical mincing, facilitates and increases the SR and CLB rates substantially in NOA patients. The combination of an adequate surgical approach, i.e. sampling multiple biopsies, together with an experienced laboratory using appropriate recovery protocols seems crucial to maximize SR. Yet, to date, it remains unclear whether there is any difference in the added value of enzymatic digestion between conventional TESE and micro-TESE in order to facilitate and maximize SR. Future research should also focus on alternative laboratory techniques for facilitating recovery, limiting the processing time of samples and selecting the best sperm for injection, in order to further improve the overall success rate of ICSI using sperm from NOA patients. Therefore, we are looking forward to the added value of artificial intelligence technology to facilitate both recovery and selection of the testicular sperm in order to improve reproductive outcomes.

## Data Availability

The data underlying this article cannot be shared publicly due to the privacy of individuals who participated in the study. The data will be shared on reasonable request to the corresponding author.
